# Aplastic anemia secondary to dual cancer immunotherapies a physician nightmare: case report and literature review

**DOI:** 10.1186/s13223-021-00616-4

**Published:** 2021-10-26

**Authors:** Romy G. Younan, Roy A. Raad, Bassem Y. Sawan, Rabih Said

**Affiliations:** 1 Medical Oncology Department, Saint George Hospital University Medical Center and University of Balamand, Beirut, Lebanon; 2 Medical Imaging Department, Saint George Hospital University Medical Center and University of Balamand, Beirut, Lebanon; 3 Pathology Department, Saint George Hospital University Medical Center and University of Balamand, Beirut, Lebanon

**Keywords:** Ipilimumab, Nivolumab, Anti-Thymocyte Globulin, ATG, Cyclosporine, Eltrombopag

## Abstract

**Background:**

Treatment with immune checkpoint inhibitors has revolutionized cancer treatment over the past several years. Despite their clinical benefits, a wide range of immune-mediated toxicities can be observed including hematological toxicities. Although, the majority can easily be managed, immune-mediated adverse events rarely can be severe and difficult to approach. Herein, we are reporting a case of very severe aplastic anemia secondary to ipilimumab (I) and nivolumab (N) treatment that failed various treatment including intensive immune suppressive therapy.

**Case presentation:**

We described a case of a 45-year old white male, heavy smoker presented to the clinic complaining of left flank pain. He was found to have a metastatic renal cell carcinoma for which he was treated with dual immunotherapy and later complicated by severe immune related adverse events. The patient later died after failing intensive immune suppressive therapy.

**Conclusion:**

Immunotherapy has become an established pillar of cancer treatment improving the prognosis of many patients with variant malignancies. Yet, lethal adverse events can occur in rare cases. It is our duty, as physicians, to remain alert and cautious.

## Background

Aplastic anemia is classified as non-severe (NSAA), severe (SAA) and very severe (vSAA) based on the degree of the peripheral blood cytopenias [1, 2]. Survival in severe aplastic anemia has markedly improved in the past 4 decades because of advances in hematopoietic stem cell transplantation, immunosuppressive biologics and drugs, and supportive care. Hematopoiesis can be restored in SAA with hematopoietic stem cell therapy (HSCT) or immunosuppressive therapy (IST). However, most patients are not suitable candidates for optimal initial HSCT because of lack of a matched sibling donor, lead time to identify suitable unrelated donor, age, comorbidities, or access to transplantation. Standard initial IST with horse anti-thymocyte globulin (ATG) and cyclosporin (CsA) can produce hematologic recovery in 60–70% of cases and excellent long-term survival among responders, as shown in several large prospective studies worldwide [3, 4]. Here we described a case of patient with metastatic renal cell carcinoma (RCC) treated with immunotherapy complicated by very severe aplastic anemia. We will discuss the challenges in diagnosis and treatment with literature review of the reported cases.

## Case presentation

**Fig. 1:**
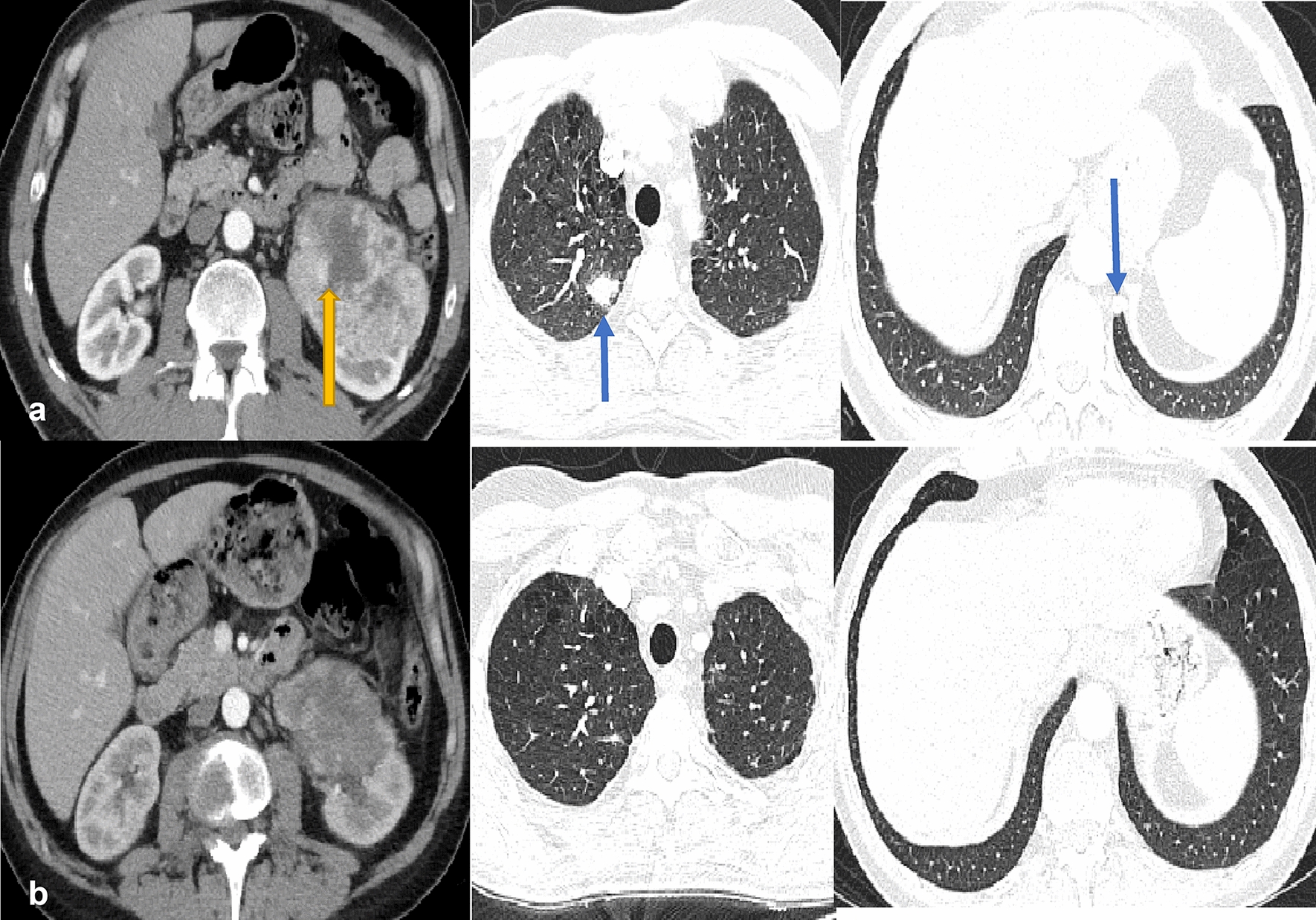
**A**, **B** Axial contrast-enhanced CT images of the abdomen (soft tissue kernel) and chest (lung kernel) obtained at baseline (**A**, May 2019) and follow-up after treatment (**B**, September 2019). Large heterogeneous enhancing left renal exophytic mass representing the known primary renal cell carcinoma (yellow arrow), decreasing in size on the follow-up examination (**B**), with resolution of the lung metastases in the right upper and left lower lobes (blue arrows). Imaging findings were consistent with favorable response to therapy

**Fig. 2:**
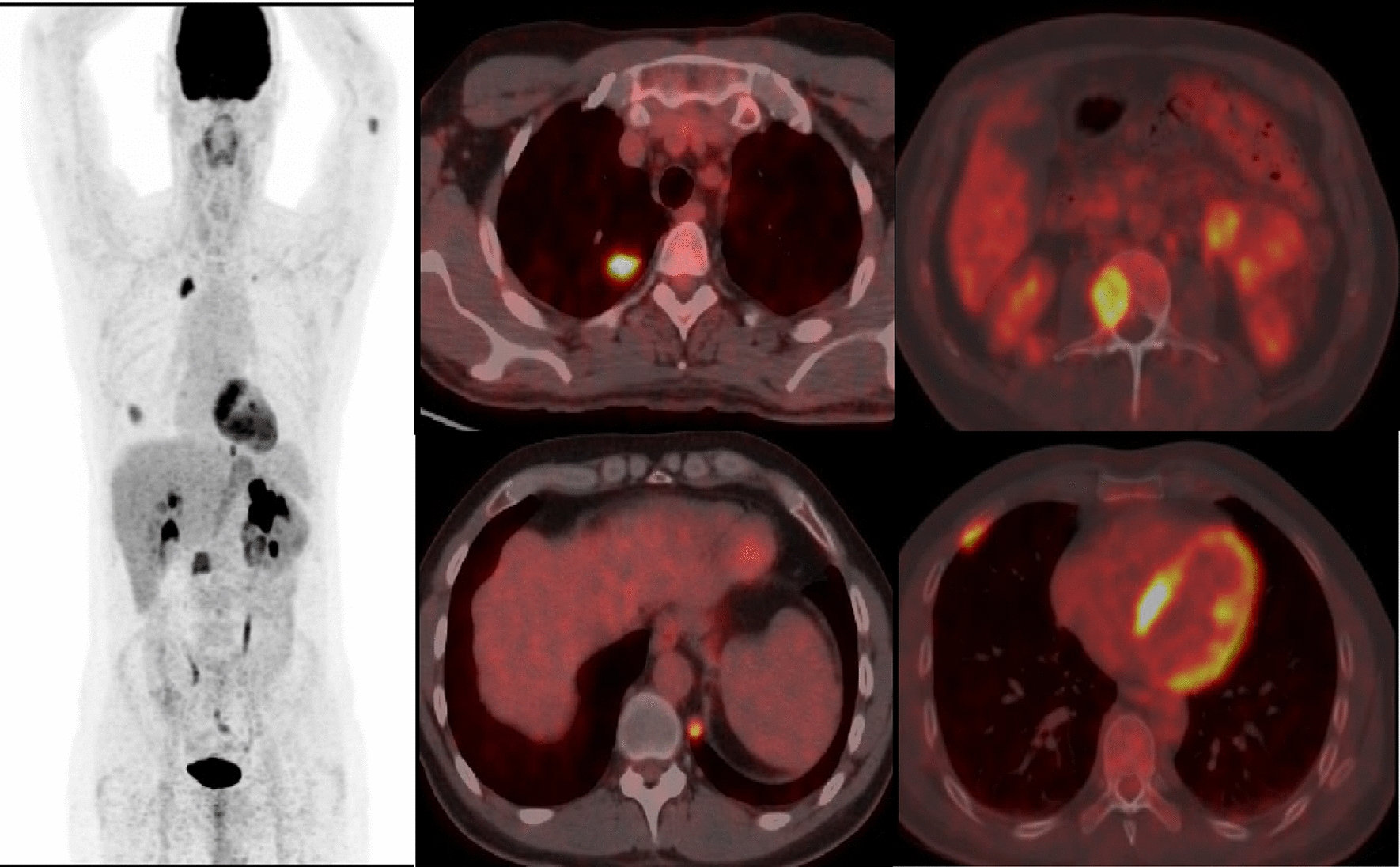
Whole-body MIP and axial fusion images from an 18F-FDG PET/CT peformed for initial staging. FDG left renal mass is seen, representing the known renal cell carcinoma. Intense uptake is seen with the previously visualized lung nodules in the right upper and left lower lobes, as well as additional FDG avid lytic osseous lesions in the right anterior 5th rib and the L3 vertebral body, consistent with distant metastases

A 45-year old male, heavy smoker presented to the clinic in May 2019 complaining of left flank pain. Chest and abdominal CTs scan revealed a suspicious enhancing left renal mass along with two worrisome lung nodules in the right upper and left lower lobes (Fig. 1A). Subsequently, ^18^F-FDG PET-CT was performed, redemonstrating the large left renal mass, with FDG avid retroperitoneal adenopathy consistent with nodal mestastases, increased FDG uptake within the lung nodules, as well as FDG avid lytic osseous lesions in the L3 vertebral body and the right anterior fifth rib, consistent with metastases (Fig. 2). The pathology results of the biopsies taken from the renal mass and one lung nodule were compatible with metastatic renal clear cell carcinoma. The patient was classified as an intermediate-risk metastatic RCC according to the IMDC criteria and therefore, he was started on a first-line dual immunotherapy treatment consisting of 1 mg/kg ipilimumab and 3 mg/kg nivolumab; each cycle every 3 weeks. He received the first cycle on June 13, 2019 and 2nd cycle on July 4th 2019.

After his 2nd cycle, the patient started to develop several immune-related adverse events (irAEs); a grade [3] immune mediated hepatitis followed by a grade [4] immune related thyroiditis. The treatment was held and he was started on oral corticosteroid therapy 1 mg/kg/day with rapid improvement of his liver function tests and subsequently cancelation of liver biopsy. Similarly, thiamazole were prescribed to resolve his thyroiditis gradually.

In August 2019, the patient, while taking tapering steroid, presented to the clinic for new onset gingival bleeding along with diffuse purpura/petechiae on his upper and lower limbs. He was awake and oriented but pale looking. His vital signs were stable. Physical exam was significant for petechia and purpura mainly on the abdomen and upper limbs. Blood work showed severe thrombocytopenia (Platelets count 15,000 mm^3^) with normal white blood cells (WBC) including differentials and Hemoglobin (Hgb). The patient was recommended to increase his steroids back to 1 mg/kg/day and he was referred to the hospital for management.

**Fig. 3:**
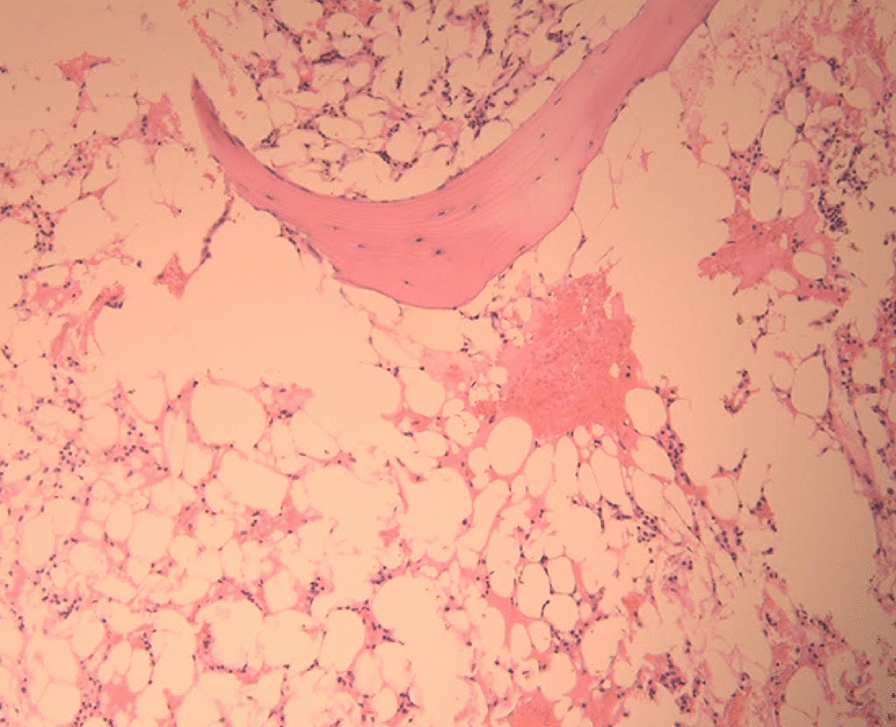
Bone marrow biopsy reveals a hypocellular marrow with global trilineage hypoplasia

Repeat blood work was remarkable for WBC 1.68 × 1000, Neutrophils 45%, Lymphocytes 53%, Hgb 10.9 g/dl, platelets 7000 mm^3^. The patient was started on higher dose steroids (2 mg/kg/day), intravenous immunoglobulin (400 mg/kg) daily for 5 days and a bone marrow biopsy was performed and confirmed the diagnosis of aplastic bone marrow (Fig. 3). The biopsy showed a markedly hypocellular marrow (< 10%) with severe trilineage hypoplasia. There was no morphological evidence of blasts excess or myelodysplasia. Metastatic tumor foci were excluded (confirmed by immunostains). Neither was there immunophenotypic evidence of elevated myeloid/lymphoid precursors or a B cell neoplasm. The lymphocyte fraction was composed of 80% T cells with an inverted CD4^+^:CD8^+^ ratio (1:2). In addition, flow cytometry showed absence of hematogones, consistent with a diagnosis of AA.

Restaging chest and abdominal CT scans showed evidence of favorable response to therapy, including decreasing size of the left renal primary tumor and disappearance of lung metastases (Fig. 1B).

During his prolonged hospital stay, the patient received multiple irradiated blood products transfusions and broad-spectrum antibiotics and antifungal therapy. In addition to above mentioned treatment, he was started on, G-CSF (1 injection daily), and eventually he received an intense triple IST: horse ATG (20 mg/kg) IV daily for 5 days along with eltrombopag (150 mg tablet/daily) and Cyclosporine (6 mg/kg/day) in two divided doses with weekly monitored trough levels**.** Unfortunately, his bone marrow failed to respond to the immunosuppressive therapy. The case was discussed with stem cell transplant team but he was not candidate due to uncontrolled RCC which started to grow again by that time. Unfortunately, the patient later died after continuous support and palliative care in October 2019.

## Discussion and conclusions

To our knowledge, this is the first case of ipilimumab and nivolumabinduced aplastic anemia that received the standard of care IST; horse ATG and CsA. Induced aplastic anemia in the setting of cancer immunotherapy is an extremely rare occurrence and has only been reported in the literature three times previously [5–7], with one case occurring secondary to single-agent nivolumab [5]. Two of these cases were fatal [5, 6]. Only one patient with mild AA was able to recover after response to steroid therapy [7].

Our patient initially presented with thyroiditis and hepatitis. Few weeks later, he presented with severe thrombocytopenia. Considering the events, our clinical and therapeutic approach was ITP in context of ipilimumab and nivolumab.

In a review of 19 large clinical trials of ICIs, such as anti-PD-L1, anti-PD-1, and anti-cytotoxic T-lymphocyte-associated antigen 4 antibodies, used for the treatment of melanoma, lung cancer, renal cancer, and bladder cancer, the frequency of the occurrence of hematological irAE was estimated to be 3.6% cases for all grades and 0.7% cases for grades 3 or 4. ITP was reported to be one of the most frequent type of hematological irAEs [8, 9]. However, as he started to have progressive pancytopenia a bone marrow biopsy was done.

Once the diagnosis of AA was confirmed, the decision to initiate IST was initiated as described. Since he had vSAA and good clinical performance, he was started on an intense triple IST: horse ATG, CsA, and eltrombopag. Survival rates following this regimen are theoretically equivalent to those achieved with HSCT. Furthermore, analysis of several clinical data suggests that patients with minimal blood count responses to a single course of ATG, even when transfusion independence is achieved, have a markedly worse prognosis than patients with robust hematologic improvement [10] which was noted in our patient.

**Fig. 4:**
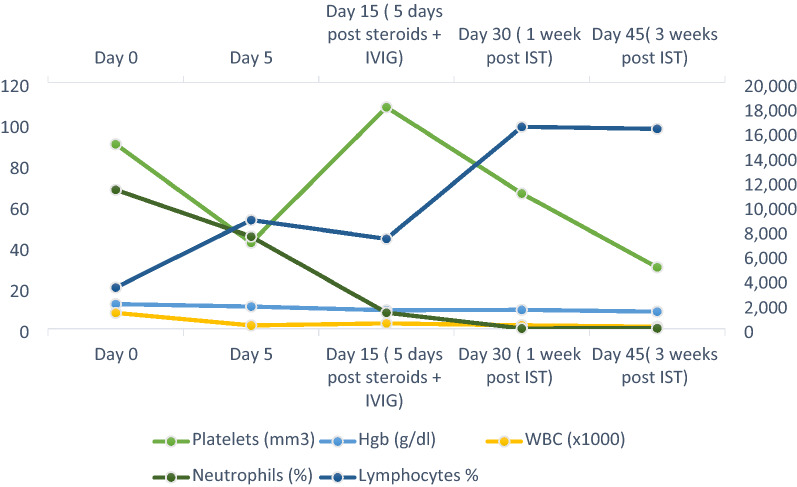
Hematological Indices Timeline. *Hgb* hemoglobin, *WBC* white blood cells

Unfortunately, few weeks after IST, our patient failed to exhibit any hematological response signs; He had no improvement in the ANC, platelet count, or reticulocyte count as outlined (Fig. 4). In addition, his RCC started to worsen and therefore, he was not found to be candidate for stem cell transplant.

In conclusion, dual immunotherapy for cancer is a successful leap in treating various tumors but it remains a double-bladed sword if severe adverse events occur despite being rare. Aplastic anemia secondary to immunotherapy remains rare; however, we, as physicians, should remain vigilant of this nightmare occurrence.

## Data Availability

The datasets generated and/or analyzed during the current study are not publicly available due to privacy of the patient’s file and archive but are available from the corresponding author on reasonable request.

## Abbreviations

I: Ipilimumab
N: Nivolumab
NSAA: Non-severe aplastic anemia
SAA: Severe aplastic anemia
vSAA: Very severe aplastic anemia
HSCT: Hematopoietic stem cell therapy
IST: Immunosuppressive therapy
AST: Anti-thymocyte globulin
CsA: Cyclosporin
RCC: Renal cell carcinoma
irAEs: Immune-related adverse events
WBC: White blood cells
Hgb: Hemoglobin
